# Electric field stimulation unmasks a subtle role for T-type calcium channels in regulating lymphatic contraction

**DOI:** 10.1038/s41598-023-42877-6

**Published:** 2023-09-22

**Authors:** Michael J. Davis, Jorge A. Castorena-Gonzalez, Scott D. Zawieja

**Affiliations:** 1 Department of Medical Pharmacology and Physiology, University of Missouri School of Medicine, One Hospital Drive, MA415 Medical Sciences Building, Columbia, MO 65212 USA; 2 Department of Pharmacology, Tulane University School of Medicine, New Orleans, LA 70112 USA

**Keywords:** Physiology, Cardiovascular biology

## Abstract

We previously identified two isoforms of T-type, voltage-gated calcium (Ca_v_3) channels (Ca_v_3.1, Ca_v_3.2) that are functionally expressed in murine lymphatic muscle cells; however, contractile tests of lymphatic vessels from single and double *Ca*_*v*_*3* knock-out (DKO) mice, exhibited nearly identical parameters of spontaneous twitch contractions as wild-type (WT) vessels, suggesting that Ca_v_3 channels play no significant role. Here, we considered the possibility that the contribution of Ca_v_3 channels might be too subtle to detect in standard contraction analyses. We compared the sensitivity of lymphatic vessels from WT and *Ca*_*v*_*3* DKO mice to the L-type calcium channel (Ca_v_1.2) inhibitor nifedipine and found that the latter vessels were significantly more sensitive to inhibition, suggesting that the contribution of Ca_v_3 channels might normally be masked by Ca_v_1.2 channel activity. We hypothesized that shifting the resting membrane potential (Vm) of lymphatic muscle to a more negative voltage might enhance the contribution of Ca_v_3 channels. Because even slight hyperpolarization is known to completely silence spontaneous contractions, we devised a method to evoke nerve-independent, twitch contractions from mouse lymphatic vessels using single, short pulses of electric field stimulation (EFS). TTX was present throughout to block the potential contributions of voltage-gated Na^+^ channels in perivascular nerves and lymphatic muscle. In WT vessels, EFS evoked single contractions that were comparable in amplitude and degree of entrainment to those occurring spontaneously. When Ca_v_1.2 channels were blocked or deleted, only small residual EFS-evoked contractions (~ 5% of normal amplitude) were present. These residual, EFS-evoked contractions were enhanced (to 10–15%) by the K_ATP_ channel activator pinacidil (PIN) but were absent in Ca_v_*3* DKO vessels. Our results point to a subtle contribution of Ca_v_3 channels to lymphatic contractions that can be unmasked in the absence of Ca_v_1.2 channel activity and when the resting Vm is more hyperpolarized than normal.

## Introduction

Collecting lymphatic vessels generate spontaneous, twitch-like contractions that propel lymph centrally, accounting for 2/3 of peripheral lymph flow during quiet standing^1,2^. These contractions are triggered by action potentials (APs) in lymphatic muscle cells (LMCs), whereby a single AP evokes a transient contraction entrained for the length of one or more lymphangions^3,4^. Although the ionic conductances underlying the AP in LMCs have not been completely resolved, inward current during the AP spike is carried by voltage-gated calcium channels (VGCCs) with a contribution of voltage-gated sodium channels (VGSCs) in some species^5–12^. T-type VGCCs are also expressed in mesenteric lymphatic vessels from rat^13^ and sheep^7^ and proposed to regulate the frequency of the ionic pacemaker driving spontaneous contractions^10,13^.

In a recent study of peripheral collecting lymphatics, we confirmed that L-type VGCCs (Ca_v_1.2, encoded by *Cacna1c* and hereafter referred to as *Ca*_*v*_*1.2*) and T-type VGCCs (Ca_v_3.1 and Ca_v_3.2, encoded by *Cacna1g* and *Cacna1h*, respectively, and hereafter referred to as *Ca*_*v*_*3.1* and *Ca*_*v*_*3.2*, respectively, or collectively as *Ca*_*v*_*3*) are expressed in LMCs of both rats and mice. We demonstrated through patch clamp protocols that products of *Ca*_*v*_*1.2* and *Ca*_*v*_*3* transcription form functional calcium channels gated by depolarization in rat and mouse LMCs^14^. However, contractile tests of lymphatic vessels from *Ca*_*v*_*3.1*^*−/−*^ mice, and *Ca*_*v*_*3.2*^*−/−*^ mice, and even *Ca*_*v*_*3.1*^*−/−*^*; Ca*_*v*_*3.2*^*−/−*^ double knock-out (DKO) mice, exhibited nearly identical parameters of spontaneous twitch contractions as wild-type (WT) control vessels (i.e., equivalent values of frequency, amplitude, ejection fraction and fractional pump flow), over a wide pressure range. Thus, although functional Ca_v_3 channels are expressed in murine lymphatic smooth muscle, we concluded that they do not play a detectable role in determining these aspects of lymphatic contraction in mice. In contrast, smooth-muscle specific deletion of *Ca*_*v*_*1.2* abolished all spontaneous contractions, confirming that Ca_v_1.2 channels are critical for their initiation and generation. While these findings may apply only to the mouse and not to other species, commonly-used concentrations of the T-channel inhibitors Ni^2+^ and mibefradil produced consistent inhibition of spontaneous contractions in lymphatic vessels from *Ca*_*v*_*3.1*^*−/−*^*;Ca*_*v*_*3.2*^*−/−*^ (*Ca*_*v*_*3* DKO) mice, suggesting that these compounds inhibit lymphatic smooth muscle primarily through their actions on Ca_v_1.2 channels and, therefore, have limited use in detecting the specific contributions of Ca_v_3 channels in LMCs. Until the development of truly Ca_v_3 selective inhibitors, genetic deletion strategies are necessitated to provide definitive answers about the contributions of Ca_v_3 channels to lymphatic contractile function.

In the present study, we developed additional tests to determine if Ca_v_3 channels play a more subtle role in lymphatic function than revealed by standard analyses of spontaneous lymphatic contractions. First, we hypothesized that Ca_v_3 channels, while insufficient to initiate lymphatic action potentials in the absence of Ca_v_1.2, may participate in the calcium entry required for full-amplitude contractions and normal pacemaking, and that progressive and selective inhibition of Ca_v_1.2 channels by a dihydropyridine antagonist might uncover a role for Ca^2+^ entry through Ca_v_3 channels. That idea was tested by comparing the contractile responses of WT and *Ca*_*v*_*3* DKO vessels to increasing concentrations of nifedipine (NIF). In WT vessels, voltage-dependent Ca^2+^ entry into LMCs should be mediated by both Ca_v_1.2 and Ca_v_3 channels, whereas in *Ca*_*v*_*3* DKO vessels, voltage-gated Ca^2+^ entry into LMCs should be mediated only by Ca_v_1.2 channels. Second, we hypothesized that shifting the LMC resting membrane potential (Vm) to a more negative voltage might unmask a contribution of Ca_v_3 channels to contractions. Because even slight hyperpolarization can eliminate spontaneous contractions^15^, we optimized methods to evoke nerve-independent, twitch contractions from mouse lymphatic vessels using an external field of depolarizing current. In WT vessels, single, short pulses of electric field stimulation (EFS) evoked single, large-amplitude contractions that were entrained along the length of the cannulated lymphatic vessel. This activity could be blocked by NIF in WT vessels and was absent in *Ca*_*v*_*1.2* KO vessels. However, in the presence of tetrodotoxin (TTX) to block VGSCs and NIF to block Ca_v_1.2 channels, small residual EFS-evoked contractions were present in WT vessels; these contractions were enhanced by the K_ATP_ channel activator pinacidil (PIN), but were absent in *Ca*_*v*_*3* DKO vessels, and thus presumably mediated by Ca_v_3 channels. The responses of WT lymphatic vessels were then compared to vessels from *Ca*_*v*_*3* DKO mice and smooth-muscle specific *Ca*_*v*_*1.2* KO mice in the presence of TTX, PIN and/or NIF. Collectively, the results of these protocols point to a subtle contribution of Ca_v_3 channels to lymphatic contraction amplitude and pacemaking frequency, that can be unmasked in the absence of Ca_v_1.2 channel activity if resting Vm is more hyperpolarized than normal.

## Results

We hypothesized that, if Ca^2+^ influx through both Ca_v_1.2 and Ca_v_3 channels is required for full-amplitude contractions and/or to maintain a normal pacemaking frequency, progressive inhibition of Ca_v_1.2 might uncover a role for Ca_v_3. Thus, we predicted that lymphatic vessels from *Ca*_*v*_*3* DKO mice would be more sensitive than WT vessels to inhibition by NIF. To test this idea, popliteal lymphatics were isolated, cannulated and allowed to establish a regular spontaneous contraction pattern at a fixed intraluminal pressure. NIF was added to the bath in cumulative concentrations from 1 nM to 10 µM, while recording spontaneous contractions for 2 min at each concentration. After the experiment, contraction amplitude (AMP) and frequency (FREQ) were determined from the diameter recording and fractional pump flow (FPF) was calculated as described in “Methods”. The data were then fit to the Hill equation (when possible) to determine the IC_50_ values from the concentration–response relationships.

**Figure 1:**
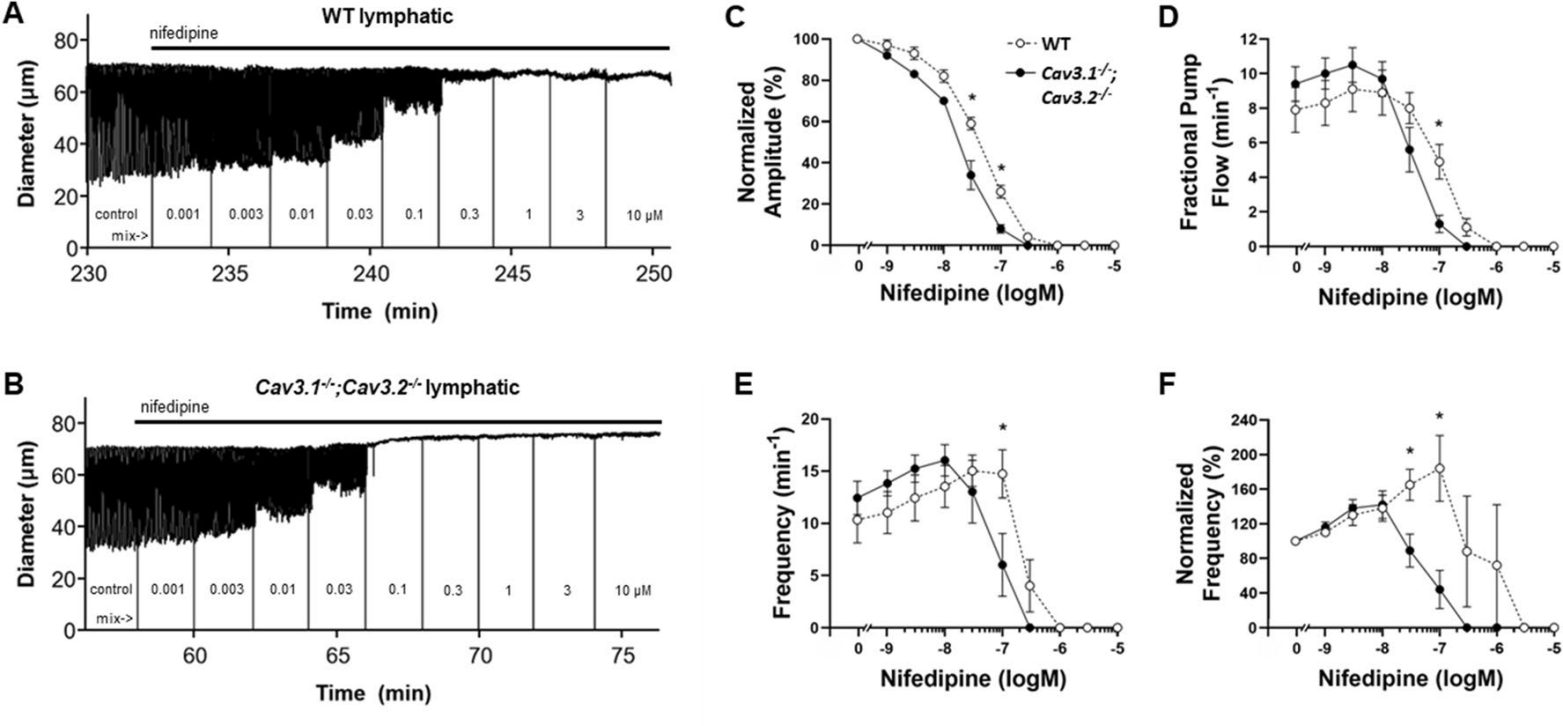
*Ca*_*v*_*3.1*^*−/−*^*;Ca*_*v*_*3.2*^*−/−*^ popliteal lymphatics are more sensitive to inhibition by NIF than WT lymphatics. (**A**) Response of a WT popliteal lymphatic vessel to increasing concentrations of NIF (applied cumulatively). Each contraction is a downward deflection (individual contractions cannot be resolved with this compressed time scale). Vertical lines are intentional artifacts created by blanking the light path to mark when a new concentration was added, followed by ~ 10 s of mixing. Pressure was held constant at 2 cmH_2_O. The cumulative DMSO concentration was < 0.4% and without effect alone. (**B**) Response of a *Ca*_*v*_*3.1*^*−/−*^*;Ca*_*v*_*3.2*^*−/−*^ popliteal lymphatic to the same NIF protocol. Contractions in the *Ca*_*v*_*3.1*^*−/−*^*;Ca*_*v*_*3.2*^*−/−*^ vessel are completely inhibited at 100 nM NIF whereas the WT vessel requires at least 300 nM NIF to block contractions. (**C**) Summary data for normalized AMP (normalized to the average AMP during the control period) as a function of NIF concentration. The curve for the *Ca*_*v*_*3.1*^*−/−*^*;Ca*_*v*_*3.2*^*−/−*^ vessels is shifted to the left by ~ 1/2 log order, with two concentrations being significantly different. Summary data for FPF (**D**) and Frequency (**E**) as a function of NIF concentration. One concentration was significantly different for each parameter. (**F**) Summary data for Normalized FREQ as a function of NIF concentration (normalized to the average FREQ during the control period). Two concentrations were significantly different and the curve for the *Ca*_*v*_*3.1*^*−/−*^*;Ca*_*v*_*3.2*^*−/−*^ vessels was shifted to the left by ~ 1 log order. Statistical tests were two-way repeated measures ANOVAs with Tukey’s multiple comparison post-hoc tests (*p < 0.05). WT: N = 5; n = 9. *Ca*_*v*_*3* DKO: N = 8; n = 15.

The results of the NIF protocol are illustrated in Fig. 1. Panels A and B show representative recordings of spontaneous contractions in popliteal lymphatic vessels from WT and *Ca*_*v*_*3* DKO mice in response to progressively higher concentrations of NIF. NIF began to inhibit AMP at about 10 nM in both vessels. NIF completely inhibited both AMP and FREQ of the *Ca*_*v*_*3* DKO vessel at 100 nM, whereas the WT vessel required a concentration of 300 nM for complete inhibition. This same pattern is evident in the summary responses shown in Fig. 1C, where there is a slight leftward-shift in the AMP-[NIF] curve for the *Ca*_*v*_*3* DKO vessels, with statistically significant differences between the normalized AMP of WT vs. *Ca*_*v*_*3* DKO vessels at 3 × 10^–8^ and 1 × 10^–7^ M NIF. Likewise, the FPF-[NIF] and FREQ-[NIF] curves for *Ca*_*v*_*3* DKO vessels were each left-shifted by ~ 1/2 log order compared to the WT curve (Fig. 1D,E), suggesting enhanced sensitivity of *Ca*_*v*_*3* DKO vessels to NIF. We also computed normalized FREQ (normalized to the initial average frequency of each vessel before NIF application) because, for concentration–response curves, this parameter is often a more sensitive indicator of a drug effect due to vessel-to-vessel variations in the basal FREQ that often occur. When FREQ was expressed as the change from control, there was ~ 1–1.5 × log left-shift in the normalized FREQ-[NIF] curve for *Ca*_*v*_*3* DKO vessels, compared to the curve for WT vessels (Fig. 1F). We repeated this protocol using popliteal lymphatics from *Ca*_*v*_*3.1*^*−/−*^ and *Ca*_*v*_*3.2*^*−/−*^ single KO mice, as shown in Suppl. Figs. 1, 2. The differences between WT and *Ca*_*v*_*3.1*^*−/−*^ vessels were more subtle than those between WT and *Ca*_*v*_*3* DKO vessels, but showed a similar trend. The FREQ-[NIF] curve for *Ca*_*v*_*3.1*^*−/−*^ vessels was left-shifted by ~ 1/2 log order (Suppl. Fig. 1E) compared to WT vessels and the normalized FREQ-[NIF] curve was left-shifted by ~ 1 log order (Suppl. Fig. 1F). *Ca*_*v*_*3.2*^*−/−*^ vessels also showed the same trend of left-shifts in AMP, FREQ, and normalized FREQ (Suppl. Fig. 2E,F), but the differences were not significantly different. The IC_50_ values for all protocols are listed in Table 1. These results are consistent with the hypothesis that Ca_v_3 isoforms may contribute to the increased sensitivity of *Ca*_*v*_*3* DKO vessels to NIF, with possibly a greater contribution from Ca_v_3.1 than Ca_v_3.2 channels.

**Table 1:** IC_50_ values for effects of NIF on spontaneous contraction parameters.

Parameter	Genotype	IC_50_ (M)	SEM of fit	Figure panel
NormAmp	WT	4.2 · 10^–8^	2.6 · 10^–9^	Figure 1C
	*Ca*_*v*_*3* DKO	1.9 · 10^–8^	2.0 · 10^–9^	Figure 1C
	*Ca*_*v*_*3.1*^*−/−*^	4.7 · 10^–8^	4.8 · 10^–9^	Suppl. Fig. 1C
	*Ca*_*v*_*3.2*^*−/−*^	3.2 · 10^–8^	3.0 · 10^–9^	Suppl. Fig. 2C
FPF	WT	1.2 · 10^–7^	1.1 · 10^–8^	Figure 1D
	*Ca*_*v*_*3* DKO	3.5 · 10^–8^	2.8 · 10^–9^	Figure 1D
	*Ca*_*v*_*3.1*^*−/−*^	7.4 · 10^–8^	4.8 · 10^–9^	Suppl. Fig. 1D
	*Ca*_*v*_*3.2*^*−/−*^	0.3 · 10^–7^	^a^	Suppl. Fig. 1D
Freq	WT	1.3 · 10^–7^	^a^	Figure 1E
	*Ca*_*v*_*3* DKO	8.8 · 10^–8^	1.3 · 10^–8^	Figure 1E
	*Ca*_*v*_*3.1*^*−/−*^	0.3 · 10^–7^	^a^	Suppl. Fig. 1E
	*Ca*_*v*_*3.2*^*−/−*^	1.0 · 10^–7^	^a^	Suppl. Fig. 1E
NormFreq	WT	9.3 · 10^–7^	5.9 · 10^–7^	Figure 1F
	*Ca*_*v*_*3* DKO	6.6 · 10^–8^	2.1 · 10^–8^	Figure 1F
	*Ca*_*v*_*3.1*^*−/−*^	6.5 · 10^–8^	^a^	Suppl. Fig. 1F
	*Ca*_*v*_*3.2*^*−/−*^	1.2 · 10^–7^	^a^	Suppl. Fig. 1F

^a^Could not be fit to Hill Equation; IC_50_ estimated from interpolated data points.

**Figure 2:**
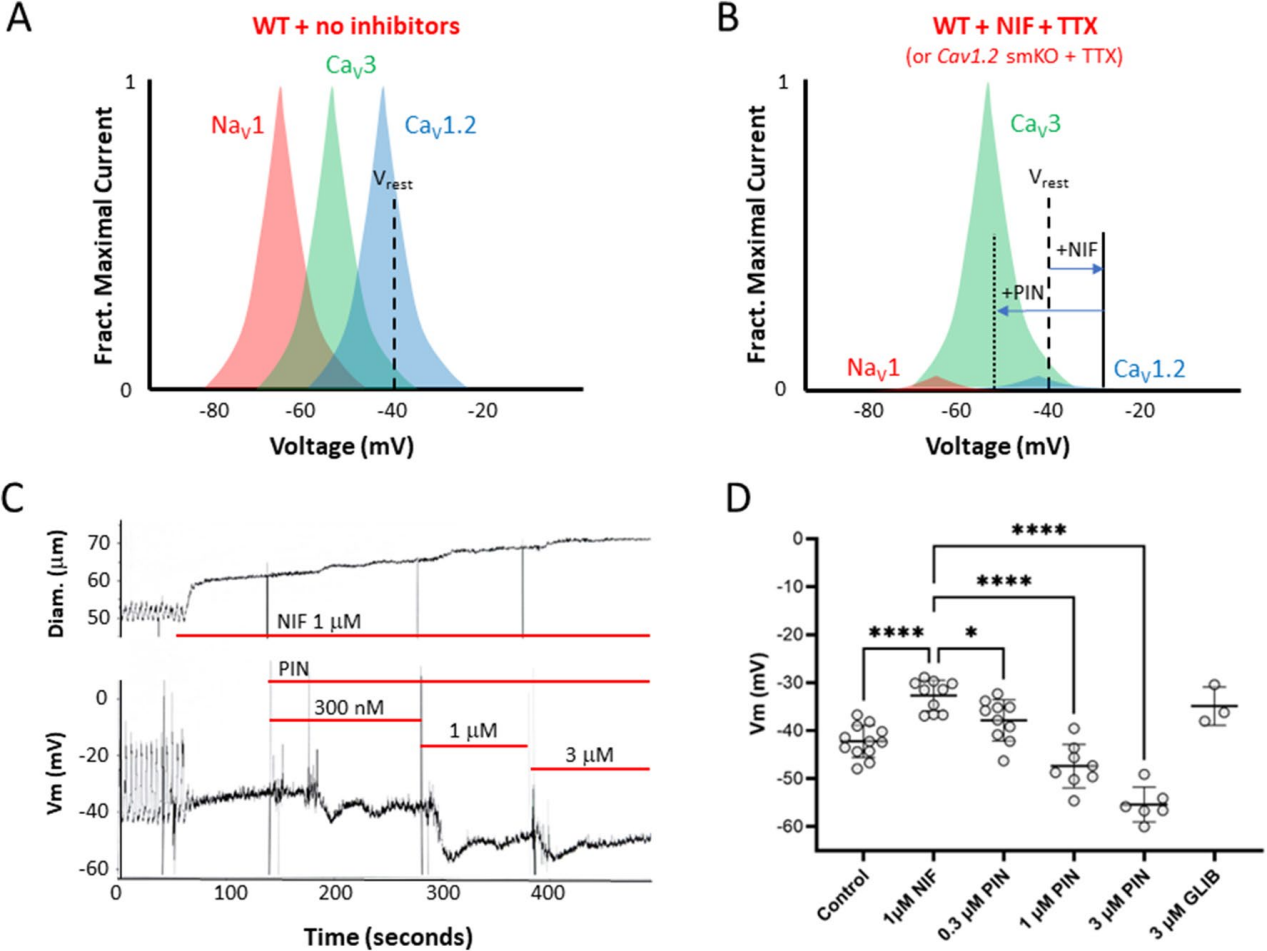
Rationale for using PIN-induced hyperpolarization to enhance *Ca*_*v*_3 current. (**A**) Theoretical depiction of window currents for Na_v_1, *Ca*_*v*_3 and *Ca*_*v*_1.2 channels relative to the resting Vm of a mouse LMC. (**B**) Depiction of the effects of NIF and PIN on Vm relative to the window currents for the three types of voltage-gated cation channels. (**C**). Experimental recording of Vm made with a sharp electrode in a mouse LMC during the application of 1 µM NIF followed by successive addition of 300 nM, 1 µM and 3 µM PIN. The vessel was treated with 2 µM wortmannin for 20 min prior to PIN application to blunt contractions (from ~ 50 to 5 µm) and reduce the chance of the electrode dislodging. NIF produced ~ 8 mV depolarization and blocked spontaneous APs. Successively higher concentrations of PIN reversed the depolarization into a net hyperpolarization. (**D**) Summary data for the effects of NIF (1 µM) followed by 300 nM, 1 µM and 3 µM PIN. GLIB (3 µM) reversed the effects of PIN. Statistical significance was determined using a mixed effects ANOVA with Tukey’s post-hoc test (*p < 0.05; **p < 0.05; ***p < 0.005; ****p < 0.005). N = 12; n = 12.

The results shown in Fig. 1, coupled with our previous negative findings^14^, raise the possibility that the contributions of Ca_v_3 channels to lymphatic contractions are too subtle to detect in standard tests of spontaneous contractions of mouse vessels. We hypothesized that a more definitive role for Ca_v_3 channels might be uncovered if the resting Vm prior to AP initiation was more hyperpolarized than normal, bringing Ca_v_3 channels more into their optimal voltage activation window. To test this, however, would require not only hyperpolarizing the membrane, but (1) inhibiting Ca_v_1.2 channels (which would otherwise predominate), (2) blocking any possible contribution from VGSC channels (likely TTX-sensitive Na_v_1 isoforms^6^), whose activity might also be enhanced at hyperpolarized potentials, and (3) evoking contractions independent of the intrinsic pacemaker, which would likely be inhibited at hyperpolarized resting potentials. The protocol depicted in Fig. 2 was devised to test our hypothesis. The theoretical window currents for Na_v_1, Ca_v_3 and Ca_v_1.2 channels, relative to the resting Vm of mouse lymphatic smooth muscle, are illustrated in Fig. 2A. At the resting Vm of the LMC, Ca_v_1.2 channels are predicted to be well within the range of their optimal window current, but Ca_v_3 channels are predicted to be barely within their range. Figure 2B depicts how this situation would change after inhibition of Na_v_1 channels^6^ by TTX and inhibition of Ca_v_1.2 channels by NIF, as the latter is known to depolarize LMCs by ~ 10 mV^12^. The subsequent addition of PIN to activate K_ATP_ channels would shift Vm to a hyperpolarized value, as we have shown recently^15^, where a greater fraction of Ca_v_3 current potentially would be available to be activated. Although the membrane likely would be too hyperpolarized to allow spontaneous activation of an AP by the intrinsic pacemaker potential, contractions potentially could be evoked by an external stimulus. An experimental recording of Vm in mouse LMCs at rest is shown in Fig. 2C, under control conditions when spontaneous APs are firing, after the application of 1 µM NIF, and after subsequent addition of PIN. Resting Vm was – 40 mV, depolarized to – 33 mV after NIF and hyperpolarized to – 40 mV after addition of 300 nM PIN, and then to ~ − 50 mV after 1 µM and 3 µM PIN. The results of several such experiments are summarized in Fig. 2D and are consistent with the NIF- and PIN-induced shifts in Vm predicted in Fig. 2B. We were unable to directly determine the amount of LMC depolarization produced by EFS because the high voltage would likely have damaged the head-stage circuitry of the amplifier during Vm measurement. The amount of PIN-induced hyperpolarization was quite variable between vessels and could be transient (see Fig. 2C and Figs. 4, 5 in Davis et al.^15^). In addition, the PIN effect on a particular vessel might be sufficient to hyperpolarize Vm out of the range for EFS-mediated depolarization. For these reasons, we tested a tenfold range of PIN (0.3 to 3 µM) on each vessel, expecting that at least one of the concentrations would produce a degree of hyperpolarization that was sufficient to recruit Ca_v_3 channels and yet still be brought to threshold by a subsequent EFS pulse.

**Figure 3:**
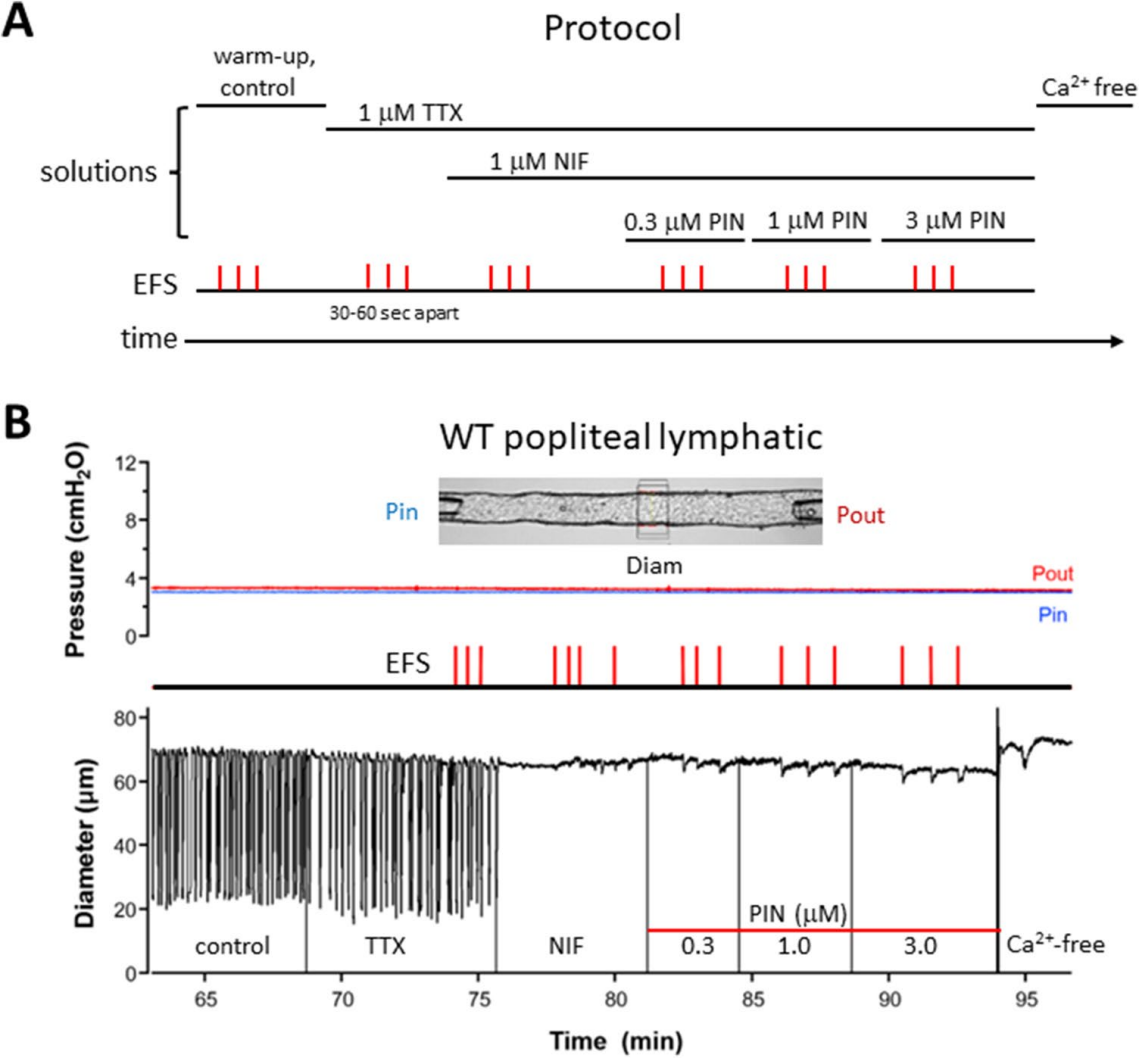
Protocol for determining the effects of PIN on amplitude of EFS-evoked contractions during inhibition of Na_v_ and *Ca*_*v*_1.2 channels. (**A**) Control measurements of spontaneous contraction amplitude were made over at least a 2-min period prior to the addition of any drug. TTX (1 µM) was applied for 5–8 min followed by NIF (1 µM) for 5–6 min prior to the addition of the three successive concentrations of PIN (2–3 min each). Three EFS pulses (0.1–0.3 ms, 80–90 V) were delivered ~ 30–60 s apart during each period of drug application. (**B**) Pressure and diameter recordings from a representative WT popliteal lymphatic to illustrate the results of the protocol. Pin and Pout are the respective inflow and outflow pressures, which were set to equal levels and held constant throughout the protocol. The bath was exchanged for Ca^2+^-free Krebs at the end of the protocol and after 30 min the maximum passive diameter was obtained for calculation of normalized diameter.

We then implemented the protocol illustrated in Fig. 3A. Single, short pulses of EFS (0.1–0.2 mS, 80–90 V) were used to elicit single contractions from WT popliteal lymphatics. The duration of the EFS pulse was set at < 0.3 mS because, when stimulus durations exceeded 1 mS, twitch contractions were often slow to recover and sometimes exhibited prolonged diastolic relaxation times and increased tone for seconds or minutes (note the contractions evoked by 1 and 5 mS pulses in Suppl. Fig. 3). Depending on the baseline contraction FREQ, pressure was lowered to 1 or 2 cmH_2_O to reduce the rate of spontaneous contractions, allowing for a sufficiently long diastolic pause in the contraction cycle, during which we could evoke an extra contraction. Thus, EFS pulses were typically delivered within a few seconds after completion of a spontaneous contraction, and we designated EFS-induced contractions as those occurring within 50 ms following an EFS pulse. The amplitudes and durations of the evoked contractions were nearly identical to those of spontaneous contractions (Fig. 3B (time = 74 min) and Suppl. Fig. 3A,B), with the timing of the EFS pulses shown) and the entrainment of each EFS-evoked contraction wave was similar to that of a spontaneous contraction, as measured from off-line analysis of the spatio-temporal (ST) maps (Suppl. Fig. 4). The ability of EFS to evoke entrained twitch contractions is in general agreement with the findings of McHale et al.^16^ in bovine mesenteric lymphatics, except for the specific values of the stimulus parameters, which are expected to vary depending on a number of factors, including the species, vessel size, chamber design, electrode diameter and placement. Contractions evoked by single EFS pulses (0.1–0.3 mS, 90 V) were not inhibited in the presence of TTX (Fig. 3B and Suppl. Fig. 3B). Curiously, the application of TTX (1 µM) in itself had almost no effect on spontaneous contraction AMP or FREQ in ~ 50% of vessels (Fig. 3B) but caused a transient slowing or cessation of spontaneous contractions in the other ~ 50% of vessels; however, the latter vessels recovered after 1–4 min and resumed a normal FREQ and AMP in the continued presence of TTX. A similar effect of TTX has been observed previously^10^; the mechanism is unknown but may reflect compensation for the contribution of Na_v_ current to diastolic depolarization by other cation channels such as Ano1^17,18^.

**Figure 4:**
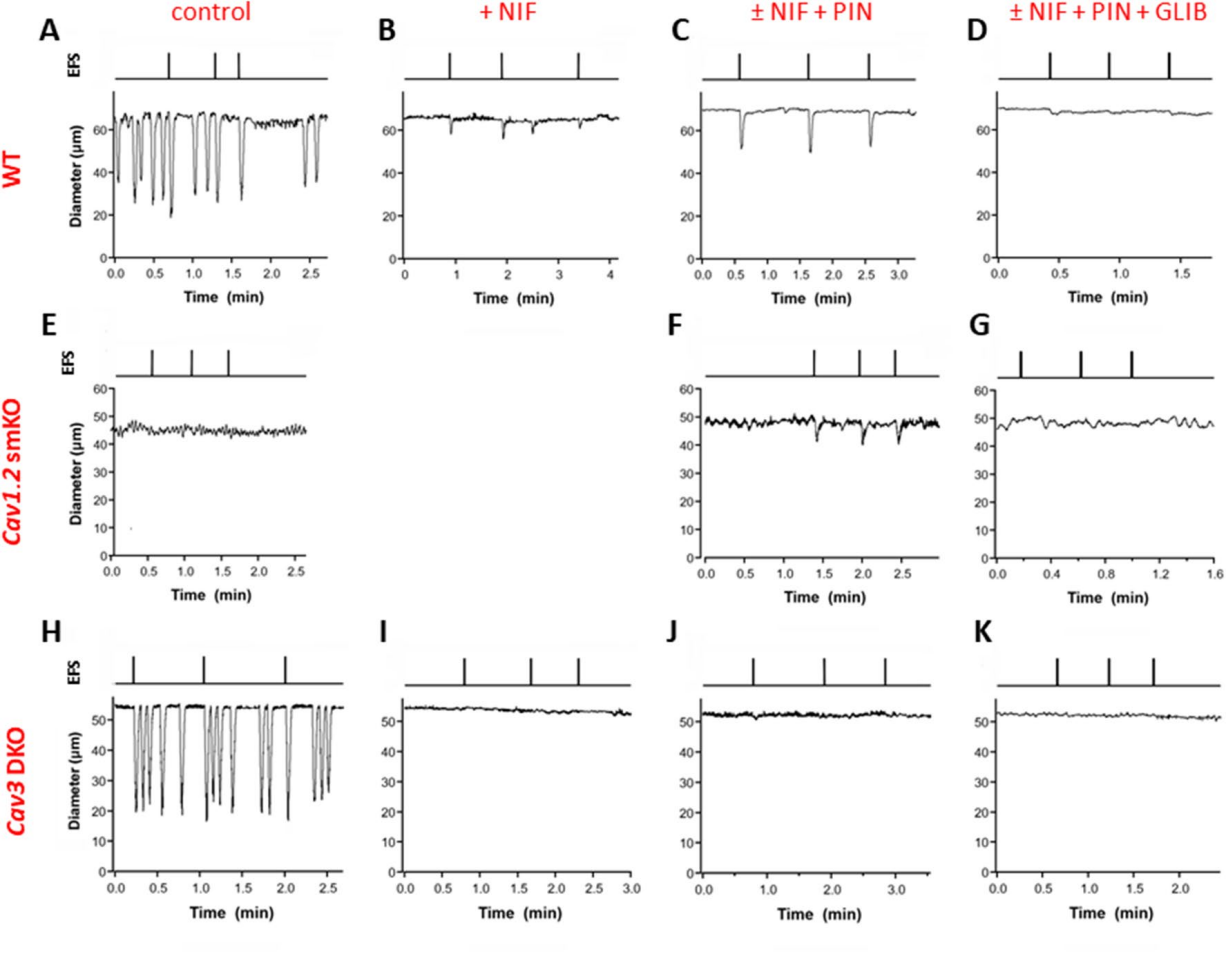
Representative recordings of EFS-evoked contractions in the three genotypes to the pharmacological treatments. (**A**–**D**) WT vessels. (**E**–**G**) *Ca*_*v*_*1.2* smKO vessels (no NIF treatment in **F** and **G**). (**H**–**K**) *Ca*_*v*_*3* DKO vessels. Note that the amplitudes of the EFS-evoked contractions were potentiated by PIN (**C** vs. **B**; **F** vs. **E**) and abolished by GLIB (**D** vs. **C**; **G** vs. **F**). TTX (1 µM) was present for all recordings. The concentrations of other compounds were NIF (1 µM), PIN (3 µM), GLIB (1 µM). All EFS pulses were 0.2 ms in duration except in A, where the duration was 0.1 ms.

Representative recordings are shown for each combination of vessel genotype and inhibitor in Fig. 4. All traces were recorded in the presence of TTX (1 µM). The set of traces at the top (Fig. 4A–D) for a WT vessel show that spontaneous contractions were blocked by NIF (1 µM) and that EFS pulses initiated only small contractions (< 5 µm in AMP; Fig. 4B). Although these contractions were much weaker than those elicited in the absence of NIF, they conducted over most of the vessel, as evident in spatio-temporal maps of the corresponding contraction waves (Suppl. Fig. 4B). The record in Fig. 4B also shows one spontaneous contraction (in between EFS pulses 2 and 3) that occurred in the presence of NIF; events of this kind exceeding 3 µm in AMP were extremely rare, were associated with much lower conduction speeds and did not consistently conduct over long distances (Suppl. Fig. 4B). The addition of PIN (3 µM), in the continued presence of NIF, resulted in larger contraction amplitudes in response to identical EFS pulses (Fig. 4C); additionally, the enhancement by PIN was blocked by 1 µM GLIB (Fig. 4D). Spontaneous contractions over 3 µm in AMP were almost never observed in vessels from *Ca*_*v*_*1.2* smKO mice (Fig. 4E). In *Ca*_*v*_*1.2* smKO vessels, EFS pulses initiated only very small or negligible contractions (Fig. 4E) that nevertheless were enhanced in AMP by PIN (Fig. 4F) and blocked by 1 µM GLIB (Fig. 4G). In contrast, vessels from *Ca*_*v*_*3* DKO mice showed spontaneous contractions with normal AMP and EFS pulses evoked additional contractions of equivalent AMP (Fig. 4H). However, in the presence of NIF (1 µM) EFS pulses failed to evoke any residual contractions in these vessels (Fig. 4I) even after the addition of PIN (Fig. 4J) or PIN + GLIB (Fig. 4K).

**Figure 5:**
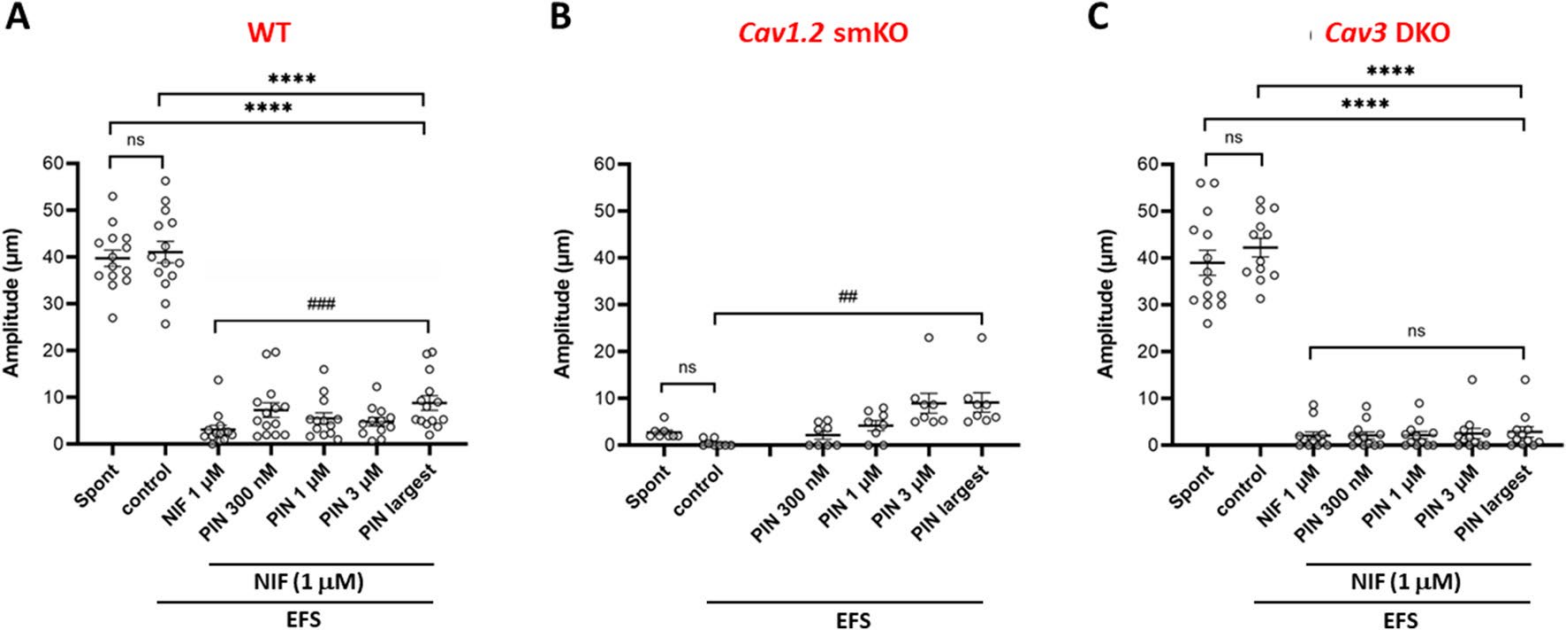
Summary data for the amplitudes of spontaneous and EFS-evoked contractions in vessels of the three genotypes during NIF and PIN treatment. (**A**) For WT vessels there were no significant differences in the amplitudes of spontaneous and EFS-evoked contractions, but the differences between both of those and the amplitudes of EFS-evoked contractions in NIF and NIF + PIN were significant (****; only the comparisons to the NIF + largest AMP of the combined PIN groups are marked). PIN significantly enhanced the AMP of EFS-evoked pulses compared to NIF alone (^###^). (**B**) Spontaneous contraction amplitudes were < 3 µm in *Ca*_*v*_*1.2* smKO vessels and EFS-evoked contractions were even smaller; the differences between the average AMP of spontaneous and EFS-evoked contractions were not significant. 1 µM and 3 µM PIN significantly enhanced the AMP of EFS-evoked contractions (^##^; only marked for the largest PIN AMP). (**C**) For *Ca*_*v*_*3* DKO vessels, there were no significant differences in the amplitudes of spontaneous and EFS-evoked contractions. However, the differences between the amplitudes of both spontaneous and EFS-evoked contractions and the amplitudes of EFS-evoked contractions in NIF and NIF + PIN were significant (****; only the comparison between the NIF + largest AMP with PIN is marked). In the presence of NIF, EFS-evoked contractions were < 3 µm in amplitude (with 2 exceptions) and were not significantly enhanced by PIN. ****p < 0.001, one-way ANOVA. ^###^p < 0.001, Wilcoxon paired signed rank test. ^##^p < 0.01, Wilcoxon paired signed rank test. Ns = not significant at p < 0.05, Wilcoxon paired signed rank test. WT: N = 7; n = 12–14. *Ca*_*v*_*1.2* smKO: N = 4; n = 9. *Ca*_*v*_*3* DKO: N = 5; n = 8.

The data for the various genotypes and pharmacological treatments are summarized in Fig. 5. TTX was present in all protocols. The amplitudes of the spontaneous contractions were averaged over a 2-min period. The amplitudes associated with EFS pulses were the averages of the contractions evoked by three pulses, excluding any cases in which a spontaneous contraction occurred too close (< 50 ms) to the EFS pulse to be certain which was the initiating event. In WT vessels, there were no significant differences between the average AMP of spontaneous contractions and the average AMP of EFS-evoked contractions (Fig. 5A). However, both were significantly different than the average AMP of EFS-evoked contractions in the presence of NIF alone or NIF plus PIN (either 300 nM, 1 µM or 3 µM, or the largest AMP of any the three PIN concentrations for each vessel). A Wilcoxon matched pairs signed rank test was used to compare the difference in the AMP of EFS-evoked contractions in NIF alone vs NIF + the largest AMP of the three PIN concentrations. This difference was highly significant, indicating that PIN significantly increased the AMP of EFS-evoked contractions when Ca_v_1.2 channels were blocked. The same analysis is presented in Fig. 5B for vessels from *Ca*_*v*_*1.2* smKO mice (but with the NIF treatment being unnecessary). In contrast to WT vessels, Ca_v_1.2-deficient vessels had extremely small spontaneous contraction amplitudes (≤ 3 µm in all but one case) and EFS pulses likewise evoked contractions with amplitudes < 1 µm. The amplitudes of EFS-evoked contractions were enhanced by all concentrations of PIN, with the difference between EFS alone and EFS+ the most effective PIN concentration (in this case always 3 µM PIN) being highly significant. Thus, the results shown in Fig. 5A and B are in agreement in showing that the amplitudes of the residual contractions evoked by EFS are enhanced when the membrane is hyperpolarized by PIN while Ca_v_1.2 channels are blocked or deleted. The PIN-induced enhancement was blocked by GLIB in WT and *Ca*_*v*_*1.2 smKO* vessels [not shown, but AMP after PIN was significantly reduced by GLIB for WT vessels (t-test; p < 0.019; n = 4) and *Ca*_*v*_*1.2* smKO vessels (t-test; p < 0.003; n = 6)]. Finally, the same analysis is shown in Fig. 5C for vessels from *Ca*_*v*_*3* DKO mice. As for vessels from WT mice, there was not a significant difference in the amplitudes of spontaneous vs. EFS-evoked contractions. However, only very small contractions (< 3 µm on average) could be evoked by EFS in the presence of NIF and these were *not* enhanced by any concentration of PIN [nor were they reduced further by GLIB (not shown; t-test; p < 0.175; n = 4)]. Nearly identical results for each of the three genotypes were produced when normalized AMP, rather than raw AMP, was used for the comparable analysis (Suppl. Fig. 5). Collectively, these results suggest that Ca_v_3 channels mediate the PIN-induced enhancement of the residual EFS-evoked contractions when Ca_v_1.2 channels are deleted or blocked.

## Discussion

In this study we asked: if functional Ca_v_3 channels are expressed in lymphatic muscle, why do they not contribute a detectable component to the AP in lymphatic muscle^14^ or make a significant contribution in the frequency or strength of spontaneous lymphatic contractions? We first examined this issue by comparing concentration–response curves for WT and *Ca*_*v*_*3* DKO vessels to the Ca_v_1.2 dihydropyridine antagonist NIF, reasoning that lymphatic vessels from *Ca*_*v*_*3* DKO mice would be more sensitive than WT vessels to inhibition by NIF because WT vessels have both Ca_v_1.2 and Ca_v_3 channels as Ca^2+^ influx sources whereas *Ca*_*v*_*3* DKO vessels have only Ca_v_1.2 channels. Vessels from *Ca*_*v*_*3*-deficient mice were indeed more sensitive to NIF (Fig. 1, Suppl. Figs. 1, 2), and the effect was more substantial for FREQ (~ tenfold more sensitive than WT) than for AMP (~ threefold). Even though the NIF concentrations (30–100 nM) associated with leftward shifts in the AMP and FREQ of *Ca*_*v*_*3* DKO vessels (Table 1) were well below those causing substantial off-target effects on Ca_v_3 channels (≤ 3 µM^14^), we could not completely rule out the possibility of off-target effects. Nevertheless, the results of that protocol were consistent with Ca_v_3 channels contributing subtly to both the AMP and FREQ of spontaneous lymphatic contractions. We then devised a second set of experiments to test for a subtle role of Ca_v_3 channels to contraction AMP, reasoning that they might be mostly inactivated under the standard conditions used previously^14^ to assess spontaneous contractions in mouse. EFS was used to initiate contractions after Ca_v_1.2 channels had been inactivated, either by NIF application or by genetic deletion of *Ca*_*v*_*1.2* from lymphatic smooth muscle. All vessels were treated with TTX to eliminate any possible contribution of Na_V_ channels whose activity could drive calcium influx through the sodium-calcium exchanger (NCX) in reverse mode. Under both conditions EFS produced small, residual contractions, 2–4 µm in AMP, compared to a normal contraction AMP of ~ 40 µm. These contractions were enhanced (to 5–10 µm, equivalent to 10–20% of the AMP of a typical, spontaneous twitch contraction) after hyperpolarizing the membrane with the K_ATP_ channel activator PIN prior to the EFS pulse. Importantly, this enhancement was absent in vessels from *Ca*_*v*_*3* DKO mice (Figs. 4, 5), confirming that the residual EFS-evoked contractions were mediated by Ca_v_3 channels. We conclude that Ca_v_3 channels make < 5% contribution to the spontaneous contraction AMP and/or FREQ of mouse lymphatic vessels under normal conditions, but that this may be enhanced by 2–threefold under conditions when the resting membrane potential is slightly hyperpolarized.

### Methodological limitations

Separating the contributions of Ca_v_1.2 and Ca_v_3 channels to Ca^2+^ influx has proven difficult in many different cell types, including LMCs. Studies of rat lymphatic vessels suggested a selective role for Ca_v_3 channels in controlling LMC pacemaking^13^, based on the effects of inhibition with mibefradil and Ni^2+^. We previously showed that two Ca_v_3 isoforms are expressed in mouse and rat LMCs and used patch clamp protocols to confirm the presence of functional channels. However, standard contraction tests revealed no significant differences between WT and *Ca*_*v*_*3* DKO vessels in either the FREQ vs. pressure or AMP vs. pressure relationships. The typical activation threshold for Ca_v_3 channels is − 20 to – 30 mV more negative than that for Ca_v_1.2 channels, and window currents for Ca_v_3 channels are similarly left-shifted^19–22^. These values are estimates from arterial SM because no comparable measurements have been made in lymphatic SM. At the resting Vm that we measure in mouse LMCs (~ − 35 mV), it is likely that Ca_v_3 channels are almost completely inactivated, unless a more right-shifted splice variant of *Ca*_*v*_*3* is expressed, as demonstrated for a left-shifted variant of *Ca*_*v*_*1.2*^23^. However, the resting Vm is slightly more negative in rat and human mesenteric LMCs (− 40 and – 45 mV, respectively^15,17^), potentially enabling more basal activity of Ca_v_3 channels in those species.

EFS was used in our experiments to override the normal LMC pacemaking mechanism so that contractions could potentially be induced without the involvement of Ca_v_1.2 channels. Although our results suggest that the residual contractions evoked by EFS are mediated by Ca_v_3 channels, EFS could also have increased Ca^2+^ influx through other smooth muscle cation channels, e.g., TRPC6, TRPM4, and/or Ca_v_2 or Ca_v_1.3 channels, all of which are resistant to NIF. Ca_v_1.3 channels in vascular smooth muscle^24,25^ are voltage-gated (but less sensitive to dihydropyridine antagonists than Ca_v_1.2^26,27^), but TRPC6 and TRPM4 channels are relatively insensitive to membrane potential^28^ and currents through those channels would not be predicted to be significantly enhanced by PIN-induced hyperpolarization. Both TRPC6 and TRPM4 are expressed in mouse LMCs (our unpublished observations), but there is no evidence for the expression of Ca_v_1.3 in lymphatic muscle, nor have we detected message for Ca_v_1.3 channels in RT-PCR assays of purified mouse LMCs or in scRNA seq assays (our unpublished observations). Importantly, the possible contributions of TRPC6, TRMP4, and other channels to the residual EFS-evoked contractions should have been the same in *Ca*_*v*_*3* DKO as in WT vessels and their potential contributions are therefore not consistent with the absence of EFS-evoked contractions in *Ca*_*v*_*3* DKO vessels (Fig. 5C).

Another possible explanation for the residual EFS-evoked contractions is that 1 µM NIF may not have completely inhibited Ca_v_1.2 channels, such that hyperpolarization prior to the EFS pulse then recruited residual Ca_v_1.2 current rather than Ca_v_3 current. Higher concentrations of NIF could have possibly blocked Ca_v_3 channels^29,30^ and it was for this reason that we also tested vessels from *Ca*_*v*_*1.2* smKO vessels. Our finding that EFS-evoked contractions in vessels deficient in *Ca*_*v*_*1.2* (Fig. 5B) were of nearly identical AMP as those in WT vessels + NIF (Fig. 5A) argues against this possibility. Additionally, PIN treatment of *Ca*_*v*_*3* DKO vessels would also have recruited whatever fraction of Ca_v_1.2 channels were not inhibited by NIF (presumably to the same degree as in WT vessels) and yet PIN did not potentiate evoked contractions under the same conditions in *Ca*_*v*_*3* DKO vessels (Fig. 5C).

It is possible that PIN could produce hyperpolarization of LMCs by acting on K_ATP_ channels in the endothelium, with the hyperpolarization being conducted to the LMC layer through myoendothelial gap junction (MEGJs). Indeed, ECs in arteries express K_ATP_ channels^31,32^ and the EC and SMC layers of arteries are electrically coupled^33^. The situation is different in lymphatic vessels, however, where LECs express only the Kir6.1 subunit and express neither SUR1 or SUR2 (Fig. 1 in Davis et al.^15^ and our unpublished sc-RNAseq data). Thus, LECs appear to lack functional K_ATP_ channels. In addition, the LEC and LMC layers have little or no electrical coupling through MEGJs^3,4^. Thus, the hyperpolarization to PIN shown in Fig. 2 was the result of direct activation of K_ATP_ channels in LMCs.

### Physiological relevance

Our results suggest that slight hyperpolarization of mouse LMCs can recruit additional Ca^2+^ influx through Ca_v_3 channels. One implication is that rat and human LMCs, in which resting Vm levels are slightly more hyperpolarized than in mouse, may normally have a larger (but probably still < 20%) contribution of Ca_v_3 channels to the AMP and/or FREQ of spontaneous contractions. This conclusion is consistent with observations of Lee et al.^13^, despite the uncertainties of the off-target effects in that study of Ni^2+^ and mibefradil on rat Ca_v_1.2 channels. Although mouse Ca_v_3 channels normally contribute < 5% to the contraction amplitude of mouse LMCs, if mouse LMCs were chronically hyperpolarized, e.g., by an endogenous or exogenous vasoactive agent, rapid depolarization to threshold would be predicted to recruit Ca_v_3 channels to participate in a subsequent AP, and potentially enhance contraction AMP and/or FREQ. This hypothesis remains to be tested.

An incidental finding from our study is that Ca_v_1.2 appears to not only mediate the upstroke of the AP in mouse LMCs (with possible contributions from Na_V_ channels), but to also modulate pacemaking frequency. The data in Fig. 1E,F, Suppl. Figs. 1E,F, 2E,F show ~ 60% rise in FREQ that occurs in response to partial inhibition of Ca_v_1.2 by low concentrations of NIF, suggesting that Ca^2+^ entry through Ca_v_1.2 channels normally retards the pacemaker. As multiple ion channels with interrelated activities comprise the currents that initiate and contribute to the LMC action potential, there are several potential mechanisms by which the sub-maximal NIF concentrations could drive increased frequency. Of note, 1 µM NIF results in a significant depolarization (Fig. 2D) and sub-maximal concentrations of NIF presumably also depolarize the cell toward threshold. Additionally, the activation of Ca_v_1.2 channels with the agonist BayK8644 results in a dramatic lengthening of the duration of the AP plateau phase^34^, whereas inhibition of Ca_v_1.2 and reduced calcium influx during the AP would be expected to accomplish the opposite, as there would be reduced activation of Ano1 and potentially of NCX. A reduction in the plateau period would shorten the overall electrical cycle and thus a higher FREQ could be achieved. Another possibility is that, while cytosolic calcium is typically considered to drive depolarization^18^, differential spatial coupling of calcium store release channels to Ano1 and Ca_v_1.2 channels vs. hyperpolarizing channels such as BK^35,36^ could provide a condition in which Ca^2+^ entry through Ca_v_1.2 channels normally retards the pacemaker.

### Clinical relevance

The relevance of Ca_v_3 channels to lymphatic function in human medicine relates to their possible therapeutic targeting to reverse contractile dysfunction of lymphatic collectors in chronic lymphedema. Olszewski's observations of patients with impaired lymphatic smooth muscle contraction strength and lower contraction frequency, or even complete loss of spontaneous contractions, in various stages of secondary lymphedema^37–39^, point to a problem involving disruption of the pacemaking mechanism that potentially can be corrected pharmacologically. However, eventual therapeutic targeting of ionic dysfunction in human lymphatic muscle will require additional insights into the specific types of ion channels involved in pacemaking, the specific isoforms of those channels expressed in humans (which may be different than in rodents), and the development of selective inhibitors to block those channels. Whether Ca_v_3 channels are expressed in human lymphatic muscle and are critical to some aspect of lymphatic function, or dysfunction, remains unknown at the present time.

## Methods

### Animal procedures

All procedures were approved by the animal care committee at the University of Missouri and complied with the standards stated in the “Guide for the Care and Use of Laboratory Animals” (National Institutes of Health, revised 2011). The study is reported in accordance with ARRIVE guidelines.

### Animals

C57BL/6J wild-type (WT) mice were purchased from Jackson Laboratory (JAX, Bar Harbor, ME, USA). *Ca*_*v*_*3.1*^*−/−*^ (*Cacna1g* null) mice on the C57BL/6J background, originally generated by Hee-Sup Shin (Korea Institute of Science and Technology^40^, were a gift from Jeffrey Molkentin (University of Cincinnati), and rederived at MMRRHC, Columbia, MO, in the C57Bl/6 background. *Ca*_*v*_*3.2*^*−/−*^ mice, originally generated by Chen et al.^41^, were obtained from JAX (B6;129-Cacna1h,tm1Kcam./J; #013770), bred into the C57Bl/6 background for at least 8 generations. *Ca*_*v*_*3.2*^*−/−*^ and *Ca*_*v*_*3.1*^*−/−*^ mice were bred to generate *Ca*_*v*_*3.1*^*−/−*^*;Ca*_*v*_*3.2*^*−/−*^ double KO mice on the C57Bl/6 background. *Myh11-CreER*^*T2*^ mice (B6.FVB-Tg(Myh11-cre/ERT2)1Soff/J), obtained from Dr. Stefan Offermanns, were bred with *Ca*_*v*_*1.2*^*f/f*^ mice (*Cacna1c*^tm3Hfm^/J; #024714), which were purchased from JAX, to generate *Myh11-CreER*^*T2*^*;Ca*_*v*_*1.2*^*l/l*^ mice (referred to as *Ca*_*v*_*1.2* smKO mice). All genotypes were verified by PCR. Mice from the latter strain were injected with tamoxifen (10 mg/ml, 100 µl i.p.) for 5 days and allowed to recover for 2 weeks before being used for experiments. Mice were provided ad libitum access to food and water and housed under normal light and dark cycles in cages of up to five mice. Mice of either sex (except for *Ca*_*v*_*1.2* smKO mice) were studied at 5–10 weeks of age (18–25 g).

### Lymphatic vessel isolation

Mice were anesthetized with pentobarbital sodium (60 mg kg^−1^, i.p.). An incision was made on the dorsal-medial side of either leg from the ankle to the groin to access the popliteal lymphatics. An excised lymphatic vessel was pinned on a Sylgard platform (Sylgard^®^ 184, Dow Corning, Midland, MI, USA) in Krebs’ buffer supplemented with 0.5% albumin, and isolated by dissection from the surrounding connective tissue and fat. After surgery, the animal was euthanized.

### Pressure myography

An excised lymphatic vessel containing at least one valve was transferred to a 3 mL chamber where it was cannulated onto two micropipettes and pressurized. The bath was exchanged at a rate of 0.5 ml/min with Krebs buffer and equilibrated for 30–60 min at 37 °C with pressure set to 3 cmH_2_O, as previously described^14^. The pipettes contained 0.5% albumin-supplemented Krebs buffer. Vessels used for further experimentation (except those from *Ca*_*v*_*1.2* smKO mice) developed robust, spontaneous contractions, with contractions that were entrained over the entire vessel length and amplitudes exceeding 30% at pressure = 3 cmH_2_O. Inner diameter at a representative region was measured continuously from video images using digital edge-detection^42^. Pressures and diameter were digitized using a National Instruments A-D system (Austin, TX) under the control of a LabVIEW program as described previously^43^.

### Sharp electrode recordings of Vm

In separate experiments, Vm was recorded in the smooth muscle cell layer of pressurized WT mouse lymphatic vessels to verify the extent of PIN-induced hyperpolarization after L-type VGCC inhibition. To permit stable recordings of Vm in contracting vessels, wortmannin (1–3 µM, 20–30 min) was used to inhibit myosin light chain kinase and blunt vessel movement; the concentration and exposure time were adjusted to preserve minimal contractions (< 5 microns) that confirmed preservation of viability. The smooth muscle layer was impaled with an intracellular microelectrode (300–350 MΩ) filled with 1M KCl, and Vm was recorded using a NPI SEC-05x amplifier (ALA instruments, Farmingdale, NY) as previously described^18^. The amplifier output was digitized and sampled at 1 kHz using an A-D interface (National Instruments). After a successful impalement, Vm was allowed to stabilize for 15–30 s. The most negative value during the AP was approximately – 35 mV. After recording multiple contraction cycles, 1 µM NIF was added to the bath solution to inhibit L-type Ca^2+^ channels. In some cases the impalement was lost due to the mixing procedure and, when that happened, attempts were made to impale the same cell or an adjacent cell and continue the protocol. Subsequently, PIN was added in cumulative concentrations (0.3, 1, 3 µM) while recording Vm. Once the recording was completed, the electrode was retracted from the cell and the recorded values were corrected for any offset potential.

### Electric field stimulation

EFS was achieved using two 0.5 mm platinum wires (Warner Instruments, #64-1942), separated by 2 mm within the 3 mL bath chamber. The wires were positioned 2 mm above the bottom of the observation chamber and insulated except for the terminal 4 mm. The cannulated vessel was positioned 1 mm from the chamber bottom, equidistant between the two wires. A Grass S48 stimulator provided the depolarizing current. Initial tests showed that single twitch contractions, of amplitude comparable to those of spontaneous contractions, could be elicited with short duration (< 1 mS), single pulses of 80–90 V. 90 V pulses were routinely used to ensure consistent responses. The synch output of the stimulator was amplified and digitized using an A–D interface (National Instr., Austin TX) to document pulse delivery in register with the diameter recording. For EFS protocols, pressure was usually set to either 1 or 2 cmH_2_O, depending on the spontaneous contraction rate, to provide a contraction pattern with a sufficiently long diastolic period to allow for single EFS pulses to be delivered in lymphatic diastole.

### Contraction wave analysis

To quantify the degree of entrainment of EFS-evoked contraction waves, brightfield videos of spontaneous contractions were acquired at video rates ranging from 30 to 50 fps. Recorded videos were then stored for offline processing, analysis, and quantification of the conduction speed. Videos of contractions were processed frame-by-frame to generate two-dimensional spatiotemporal maps (STMs) representing the measurement of the outside diameter (encoded in 8-bit grayscale) over time (horizontal axis) at every position along the vessel (vertical axis). All video processing and analyses were performed using a set of custom-written Python programs), as described previously^3^. Conduction speed was determined for each wave by the slope of the corresponding band on the ST map (by linear fit of the points defining the leading edge) and the speeds were averaged for all the contractions in a given video.

### Experimental protocols

After a vessel established a consistent pattern of spontaneous contractions, one of two protocols was conducted.

The first protocol assessed the concentration-dependent inhibition by NIF on spontaneous contractions. After equilibration and establishment of a consistent pattern of spontaneous contractions at constant pressure, bath perfusion was stopped and NIF was added in cumulative concentrations (1 nM to 10 µM) to the bath. Pressure was set at either 1 or 2 cmH_2_O, depending on the spontaneous contraction rate of a given vessel. Contraction responses were recorded for 2–3 min before the next concentration was applied and the protocol was completed within 20 min, a time period found previously not to produce significant effects on contraction FREQ or AMP due to bath evaporation.

For the second protocol, single voltage pulses (typically 0.1–0.3 mS, 90 V) were applied during the diastolic phase of the contraction cycle, with the pulses delivered 30–60 s apart and timed to produce minimal disruption to the spontaneous contraction pattern; this was repeated 3 times. With pressure maintained at 3 cmH_2_O, the bath perfusion was stopped and TTX (1 µM) applied. After assessing the effect of TTX on the contraction pattern for 3–4 min, three identical stimulus pulses were again delivered (30–60 s apart). For WT vessels, NIF (1 µM) was subsequently added to the bath and after 4 min the stimulus pulses were repeated. In a similar set of tests, vessels from *Ca*_*v*_*1.2* smKO mice were used in lieu of NIF treatment. In both cases the K_ATP_ channel activator, PIN, was then added to the bath in increasing concentrations (0.3, 1, 3 µM) to hyperpolarize LMCs, allowing 2–3 min equilibration at each concentration before delivering stimulus pulses. In some vessels the K_ATP_ channel inhibitor glibenclamide (GLIB) was then added and the response to EFS retested. Each time a drug was added to the bath the light path was temporarily blocked to create a vertical blanking artifact on the diameter trace. Tests using the same protocol were conducted on vessels from *Ca*_*v*_*3.1*^*−/−*^*;Ca*_*v*_*3.2*^*−/−*^ mice. In each case the total protocol was completed in less than 20 min.

At the end of either protocol, the vessel was equilibrated for 30 min in Ca^2+^-free Krebs buffer solution containing 3 mM EGTA and the passive diameters at 1, 2 and 3 cmH_2_O were measured. Once an experiment was complete, internal diameter traces of spontaneous contractions were analyzed off-line using custom-written LabVIEW programs to detect end diastolic diameter (EDD), end systolic diameter (ESD), and contraction frequency (FREQ), each computed on a contraction-by-contraction basis and averaged over a 2–5 min period. In a few cases where diameter tracking was noisy or inaccurate, the diameter was retracked during replay of 30-fps bright-field videos taken during the experiment. The diameter data were used to calculate commonly reported parameters that characterize the contractile function of lymphatic vessels:

1 $$\text{Amplitude (AMP)= EDD-ESD}$$

2 $$\text{Normalized AMP= }\left(\frac{\text{EDD-ESD}}{{\text{D}}_{\text{MAX}}}\right) \times {100}$$

3 $$\text{Ejection Fraction (EF)= }\left[\frac{{\text{EDD}}^{2}-{\text{ESD}}^{2}}{{\text{EDD}}^{2}}\right]$$

4 $${\text{Fractional}}\;{\text{Pump}}\;{\text{Flow}} \, \left( {{\text{FPF}}} \right) = {\text{EF}} \cdot {\text{FREQ}}$$

where D_MAX_ represents the maximum passive diameter (obtained after incubation with calcium-free Krebs solution) at the intraluminal pressure used in the protocols.

### Solutions and chemicals

Krebs buffer contained (in mM): NaCl, 146.9; KCl, 4.7; CaCl_2_, 2; MgSO_4_, 1.2; NaH_2_PO4⋅H_2_O, 1.2; NaHCO_3_, 3; Na-HEPES, 1.5; d-glucose, 5 (pH 7.4, 37 °C), equilibrated with room air. All chemicals were obtained from Sigma (St. Louis, MO, USA), except for BSA (US Biochemicals; Cleveland, OH, USA), MgSO_4_, HEPES (Fisher Scientific; Pittsburgh, PA, USA), TTX (Alomone, Israel). TTX was dissolved in citrate buffer (1 mM). NIF, PIN and glibenclamide (GLIB) were dissolved in DMSO at stock concentrations of 1 or 10 mM.

### Data analysis

Data were collected and analyzed using LabVIEW (National Instruments, Austin TX), Excel (Microsoft, Redmond, WA) and Prism 8 (Graphpad, La Jolla, CA, USA). Original recordings were plotted in IGOR (Wavemetrics, Oswego, OR). IC_50_ values were determined in Prism or IGOR. The four standard tests in Prism for normality (Anderson–Darling, D’Agostino & Pearson, Shapiro–Wilk, Kolmogorov–Smirnov) were used to evaluate each data set and revealed that at least half of the data sets were not normally distributed. Subsequently, one-way ANOVAs with Krusal–Wallis post-hoc tests were performed to compare the amplitude of spontaneous and EFS-induced contractions across pharmacological treatments for each genotype, and Wilcoxon matched pairs signed rank tests were used to compare pairs of data sets within each genotype. The specific tests used for each protocol are indicated in the figure legends. The data are expressed as mean ± standard error of the mean. P values < 0.05 were considered statistically significant, but other significance levels are marked when appropriate. N refers to the number of animals and *n* refers to the number of vessels or cells included per group.

### Supplementary Information

Supplementary Figure 1.

Supplementary Figure 2.

Supplementary Figure 3.

Supplementary Figure 4.

Supplementary Figure 5.

## Data Availability

Data are available on request from the authors. The data that support the findings of this study are available from the corresponding author upon reasonable request. Some data may not be made available because of privacy or ethical restrictions.

## Abbreviations

VGCC: Voltage-gated calcium channel
VGSC: Voltage-gated sodium channel (Na_v_)
Ca_v_3: T-type VGCC
Ca_v_1.2: L-type VGCC
TTX: Tetrodotoxin
KO: Knock-out
DKO: Double knock out
WT: Wild-type
LMC: Lymphatic muscle cell
LEC: Lymphatic endothelial cell
MEGJs: Myoendothelial gap junctions
NIF: Nifedipine
PIN: Pinacidil
GLIB: Glibenclamide
EFS: Electric field stimulation
SM: Smooth muscle
Ano1: Anoctamin 1 (calcium-activated chloride channel)
NCX: Sodium-calcium exchanger
K_ATP_: ATP-sensitive K^+^ channel
TPPM4: Member of melastatin family of transient receptor potential cation channels
TRPC6: Member of canonical family of transient receptor potential cation channels
BK: Large-conductance (big), calcium-activated potassium channel
AP: Action potential
Vm: Membrane potential
