# Supplementary figures and images for: Electric field stimulation unmasks a subtle role for T-type calcium channels in regulating lymphatic contraction
Source: Sci Rep. 2023 Sep 22;13:15862. doi: 10.1038/s41598-023-42877-6 (PMC10516884; doi:10.1038/s41598-023-42877-6)

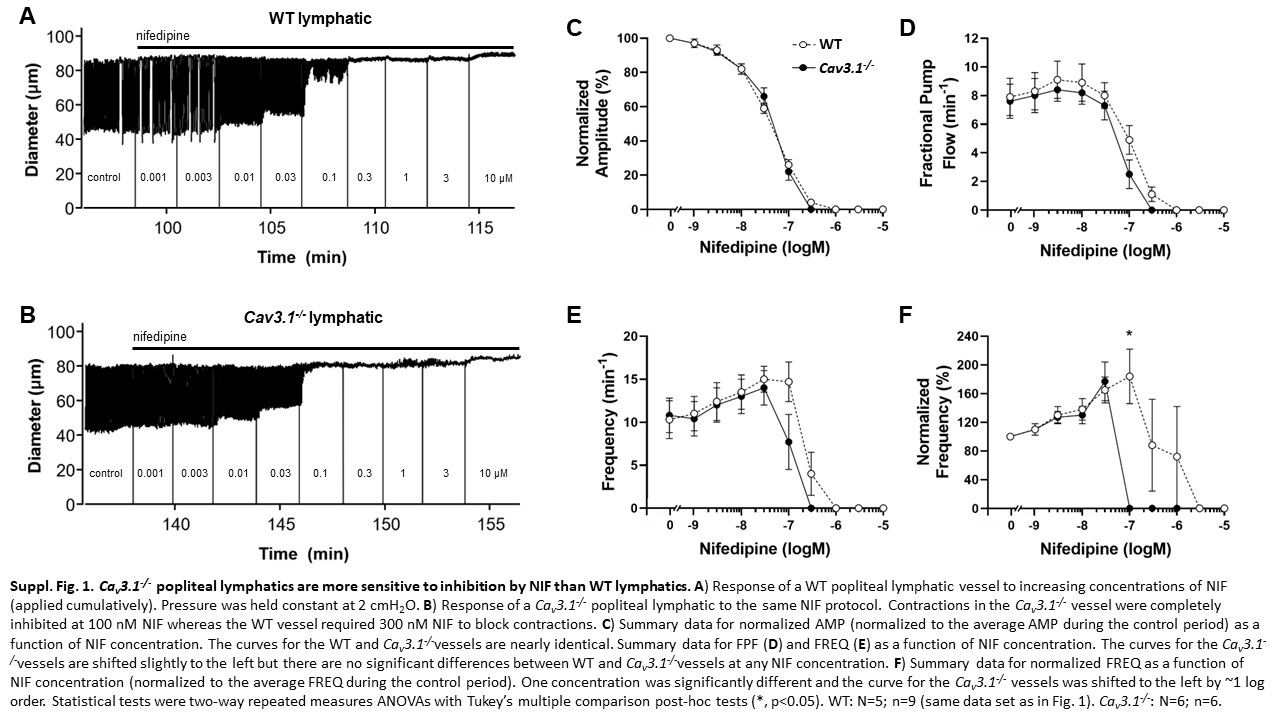

Supplement: Supplementary file 1 — Supplementary Figure 1. [file 41598_2023_42877_MOESM1_ESM.tif]

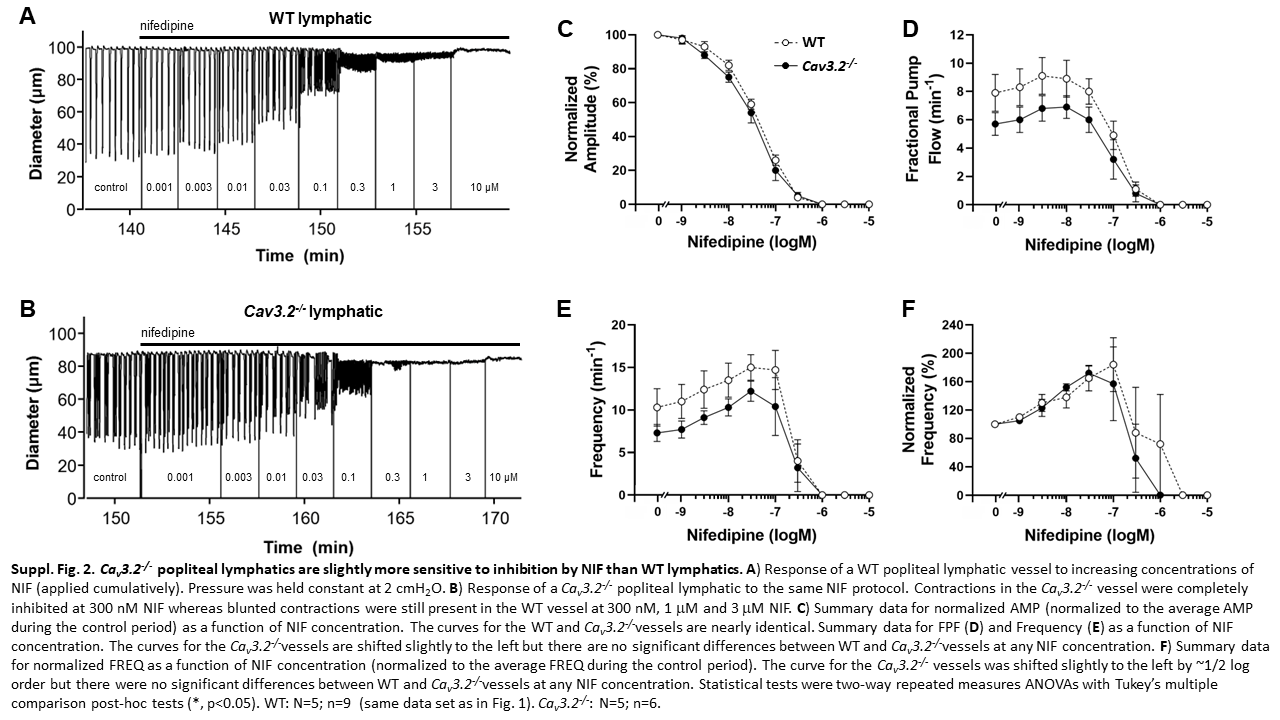

Supplement: Supplementary file 2 — Supplementary Figure 2. [file 41598_2023_42877_MOESM2_ESM.tif]

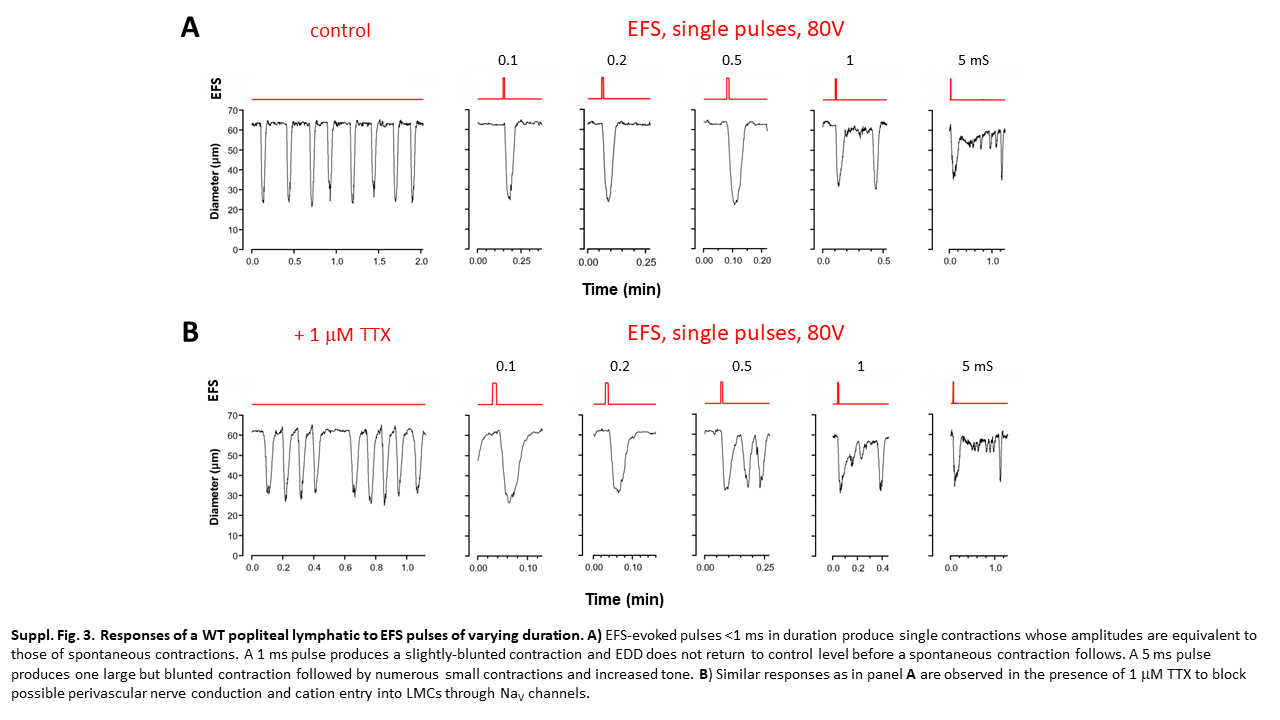

Supplement: Supplementary file 3 — Supplementary Figure 3. [file 41598_2023_42877_MOESM3_ESM.tif]

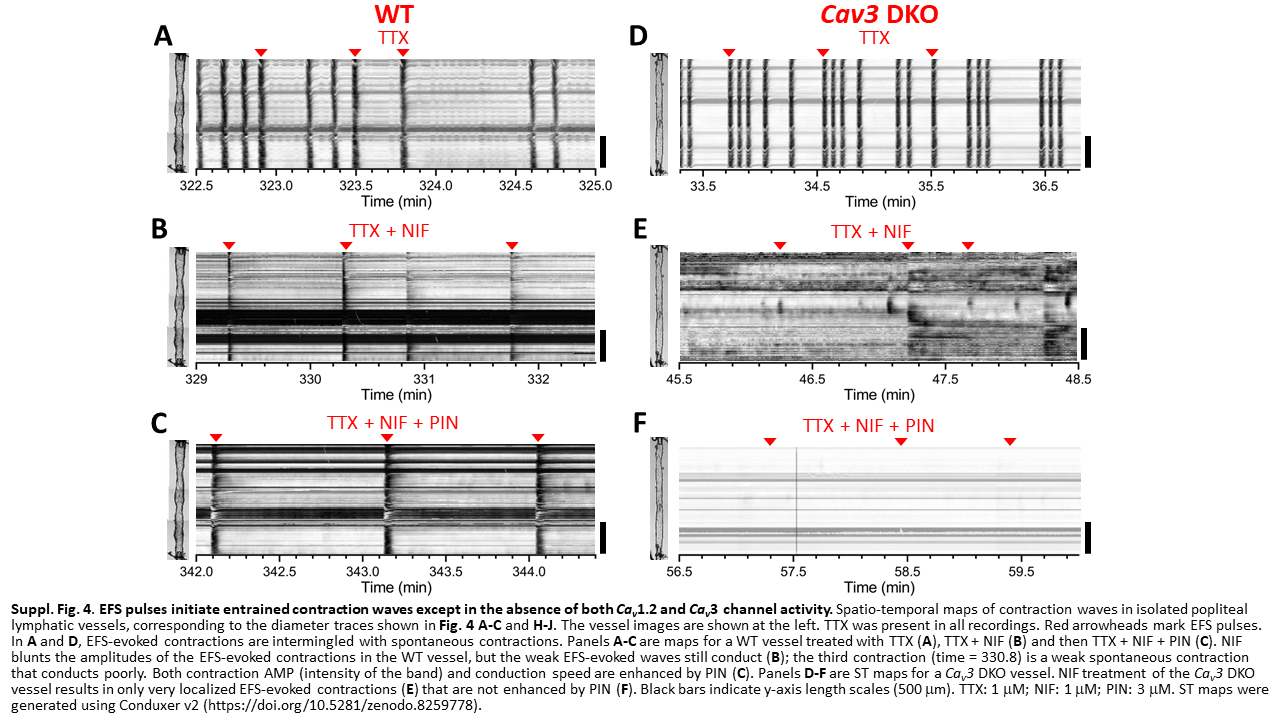

Supplement: Supplementary file 4 — Supplementary Figure 4. [file 41598_2023_42877_MOESM4_ESM.tif]

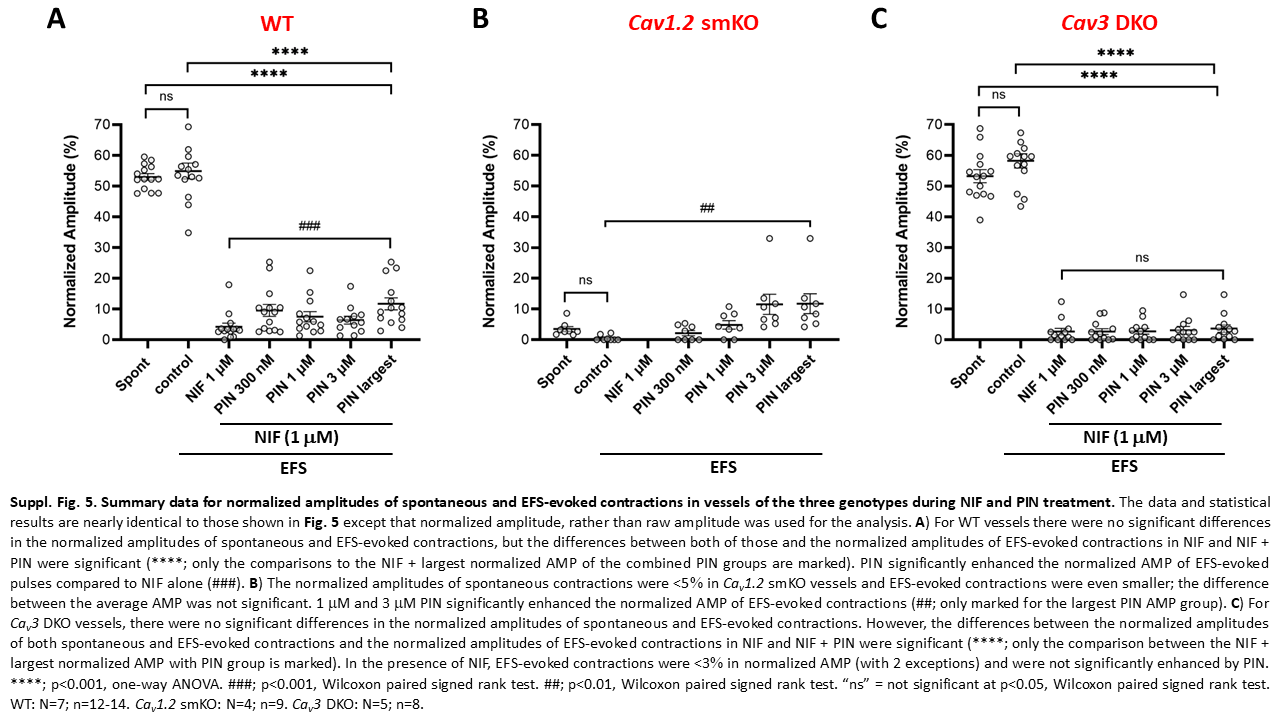

Supplement: Supplementary file 5 — Supplementary Figure 5. [file 41598_2023_42877_MOESM5_ESM.tif]
